# Conditional Deletion of *Foxg1* Alleviates Demyelination and Facilitates Remyelination *via* the Wnt Signaling Pathway in Cuprizone-Induced Demyelinated Mice

**DOI:** 10.1007/s12264-020-00583-7

**Published:** 2020-10-05

**Authors:** Fuxing Dong, Dajin Liu, Feiyu Jiang, Yaping Liu, Xiuxiang Wu, Xuebin Qu, Jing Liu, Yan Chen, Hongbin Fan, Ruiqin Yao

**Affiliations:** 1 Department of Cell Biology and Neurobiology, Xuzhou Key Laboratory of Neurobiology, Jiangsu Key Laboratory of New Drug Research and Clinical Pharmacy, Xuzhou Medical University, Xuzhou, 221004 China; 2 Public Experimental Research Center, Xuzhou Medical University, Xuzhou, 221004 China; 3 Department of Neurology, Affiliated Hospital of Xuzhou Medical University, Xuzhou, 221006 China

**Keywords:** *Foxg1*, Oligodendrocyte precursor cells, Demyelination, Remyelination, Wnt

## Abstract

The massive loss of oligodendrocytes caused by various pathological factors is a basic feature of many demyelinating diseases of the central nervous system (CNS). Based on a variety of studies, it is now well established that impairment of oligodendrocyte precursor cells (OPCs) to differentiate and remyelinate axons is a vital event in the failed treatment of demyelinating diseases. Recent evidence suggests that *Foxg1* is essential for the proliferation of certain precursors and inhibits premature neurogenesis during brain development. To date, very little attention has been paid to the role of *Foxg1* in the proliferation and differentiation of OPCs in demyelinating diseases of the CNS. Here, for the first time, we examined the effects of *Foxg1* on demyelination and remyelination in the brain using a cuprizone (CPZ)-induced mouse model. In this work, 7-week-old *Foxg1* conditional knockout and wild-type (WT) mice were fed a diet containing 0.2% CPZ w/w for 5 weeks, after which CPZ was withdrawn to enable remyelination. Our results demonstrated that, compared with WT mice, *Foxg1*-knockout mice exhibited not only alleviated demyelination but also accelerated remyelination of the demyelinated corpus callosum. Furthermore, we found that *Foxg1* knockout decreased the proliferation of OPCs and accelerated their differentiation into mature oligodendrocytes both *in vivo* and *in vitro*. Wnt signaling plays a critical role in development and in a variety of diseases. GSK-3β, a key regulatory kinase in the Wnt pathway, regulates the ability of β-catenin to enter nuclei, where it activates the expression of Wnt target genes. We then used SB216763, a selective inhibitor of GSK-3β activity, to further demonstrate the regulatory mechanism by which *Foxg1* affects OPCs *in vitro*. The results showed that SB216763 clearly inhibited the expression of GSK-3β, which abolished the effect of the proliferation and differentiation of OPCs caused by the knockdown of *Foxg1*. These results suggest that *Foxg1* is involved in the proliferation and differentiation of OPCs through the Wnt signaling pathway. The present experimental results are some of the first to suggest that *Foxg1* is a new therapeutic target for the treatment of demyelinating diseases of the CNS.

## Introduction

In the central nervous system (CNS), oligodendrocytes (OLs) are myelin-forming glial cells that play vital roles in rapid impulse conduction and normal axonal functions. Current studies have demonstrated that inflammatory stimuli or immune attacks can damage the myelin sheath, leading to OL death and myelin sheath loss, such as that observed in multiple sclerosis (MS), the most common demyelinating disease [1]. It has been reported that in early MS lesions, neural stem cells (NSCs) rooted in the region of the lateral ventricle-subventricular zone migrate, proliferate, and differentiate into oligodendrocyte progenitor cells (OPCs) induced by the injury signal of the myelin sheath and later differentiate to myelinating OLs to repair the damaged myelin [2]. However, OPCs are slow-cycling cells, and only long-term nutritional factors can stimulate their rapid proliferation and differentiation [3]. Furthermore, the repair ability of endogenous remyelination is limited, resulting in difficulties in the regeneration/repair of the myelin sheath in demyelinating diseases. Therefore, elucidating the regulatory mechanism of the correct differentiation of OPCs is crucial for understanding the myelination and remyelination processes in the CNS and is also fundamental to the treatment of demyelinating diseases [4].

*Foxg1,* also referred to as brain factor-1, is an important member of the Forkhead box transcription factor family, which plays dual roles in transcriptional activation and transcriptional repression [5]. *Foxg1* is widely expressed in the cerebral cortex and is critical to the development of the CNS [6]. One or two homologous chromosome *Foxg1* gene translocations, deletions, or mutations are closely related to various diseases associated with the occurrence and development of the CNS [7, 8]. Previous studies have shown that loss of function or downregulation of *Foxg1* reduces the proliferation of neural precursor cells, accompanied by early differentiation into neurons [9, 10]. In addition, Tian *et al*. found that *Foxg1* deletion increases the numbers of neurons and astrocytes at an early stage after birth in conditional *Foxg1* gene-knockout mice, and they speculated that *Foxg1* might inhibit neurogenesis and glial cell formation in the early stage after birth [11]. However, the regulation of *Foxg1* in the development and differentiation of OPCs is still uncertain.

The Wnt signaling pathway plays an important role in the development and myelination of OPCs [12, 13]. GSK-3β, a key kinase in the Wnt signaling pathway, negatively regulates the entry of catenin and then affects the expression of Wnt target genes [14]. Recent studies have demonstrated that GSK-3β activation promotes OPC differentiation, which may be mediated by the accumulation of nuclear β-catenin [15]. Nevertheless, whether the effect of *Foxg1* on the development and maturation of OPCs is associated with the Wnt signaling pathway has not been reported.

To investigate the role of *Foxg1* in the proliferation and differentiation of oligodendrocyte lineage cells and myelin regeneration, we conditionally knocked out *Foxg1 in vivo* to assess the loss and repair of myelin during demyelinating injury in mice, and we further explored its regulatory mechanism *in vitro*. Our findings revealed a previously-unknown function of *Foxg1* in oligodendrocyte lineage cells, suggesting that downregulating the expression of *Foxg1* in the treatment of demyelinating CNS diseases may alleviate demyelination and promote remyelination, thus providing a new gene therapy target for demyelinating CNS diseases such as MS.

## Materials and Methods

### Animals

*Foxg1* cKO mice (1 male Nestin-CreER^TM^; *Foxg1*^*fl/fl*^ mouse; 2 female *Foxg1*^*fl/fl*^ mice) were donated by Professor Chunjie Zhao from the Medical School of Southeast University. Wild-type ICR mice were purchased from the Experimental Animal Center of Xuzhou Medical University. All mice were housed with free access to food and water under a 12/12-h dark/light cycle and specific pathogen-free conditions. For *Foxg1* conditional disruption in neural stem/progenitor cells (NSPCs), Nestin-CreER^TM^ mice were crossed with *Foxg1*^*fl/fl*^ mice and induced with tamoxifen (TM; Sigma-Aldrich, St. Louis, MO, USA). The genotypes of all mice were determined by PCR analysis of tail genomic DNA with appropriate primers. Male and female mice were used for all experiments without bias. All experiments were performed according to the guidelines approved by Xuzhou Medical University Experimental Animal Ethics Committee.

The TM was dissolved in corn oil to a concentration of 10 mg/mL by shaking the solution for 3 h at 37°C. For CreER^TM^-mediated recombination, TM was intraperitoneally injected into mice at 1 mg/20 g body weight [16, 17] 3 times on alternate days.

### Experimental Model

Experimental demyelination was induced by feeding 7-week-old male mice 0.2% (w/w) cuprizone (CPZ, Sigma Aldrich, St. Louis, MO, USA) mixed into ground standard rodent chow [18, 19]. The main ingredients in the normal diet for mice in this experiment were crude protein (≥180 g/kg), crude fat (≥40 g/kg), and crude fiber (≤ 50 g/kg). These mice were fed CPZ for 5 weeks and then allowed to remyelinate for 2 weeks with normal food.

### Experimental Groups

Depending on the experimental purpose, mice were sacrificed at different times as follows: (1) to assess the knockout efficiency of *Foxg1*, 8 wild-type mice (WT group) and 8 *Foxg1* cKO mice (cKO group) were sacrificed on day 7 after TM injection; (2) at the end of 5 weeks of CPZ administration, 32 mice in 4 groups (WT, WT+CPZ, cKO, and cKO+CPZ; 8 mice per group) were sacrificed to explore the effects of *Foxg1* on CPZ-induced demyelination and OPC differentiation; (3) at the end of 4 weeks of CPZ administration, 24 mice in 3 groups (WT, WT+CPZ and cKO+CPZ; 8 mice per group) were sacrificed to explore the effects of *Foxg1* on OPC proliferation; and (4) another 24 mice from the same groups (8 mice per group) were sacrificed to investigate the effects of *Foxg1* on remyelination after 5 weeks of CPZ administration and another 2 weeks of recovery with normal chow. All mice received the same amount of chow each day.

In the OPC proliferation study, the mice were intraperitoneally injected with 5-bromo-2’-deoxyuridine (BrdU, Sigma-Aldrich, St. Louis, MO, USA) (50 mg/kg, 10 mg/mL dissolved in normal saline) twice a day for 7 consecutive days from week 3 to week 4, and then sacrificed (Fig. 3A). To assess OPC differentiation, mice were fed a CPZ-containing diet for 1 week after BrdU administration for 7 consecutive days (Fig. 4A).

### Morris Water Maze

The Morris water maze test (MWM) was used to evaluate spatial location learning and memory as described in previous studies [20, 21]. All data were recorded using a computerized video system (EthoVision 3.1, Noldus Instruments, Wageningen, Gelderland, the Netherlands).

### Histology and Immunofluorescence

Luxol fast blue staining and degrees of demyelination in three non-serial sections from each mouse were assessed semi-quantitatively in a blinded manner, as described previously [22]. The demyelination was scored as follows: 0, none; 1, rare foci; 2, a few areas; and 3, large (confluent) areas of demyelination.

For immunofluorescence, the brain sections were blocked with 5% bovine serum albumin (BSA) and 0.3% Triton X-100 (KeyGen Biotech, Nanjing, Jiangsu, China) in 0.01 mol/L phosphate-buffered saline (PBS) for 30 min at 37°C, followed by incubation with primary antibodies overnight at 4°C. The primary antibodies used were anti-CNPase (1:200, Santa Cruz, Dallas, Texas, USA), anti-MAG (1:300, Santa Cruz), anti-Nestin (1:500, Abcam, Cambridge, UK), anti-MBP (1:800, Abcam), and anti-BrdU (1:500, Abcam), anti-O4 (1:200, Sigma-Aldrich, St. Louis, MO, USA), anti-Foxg1 (1:500, Sigma-Aldrich), and anti-NG2 (1:200, Millipore, Bedford, MA, USA). The sections were treated with the appropriate FITC- or TRITC-conjugated secondary antibodies (1:200, Abcam) overnight at 4°C and then counterstained with 4’,6-diamidino-2-phenylindole (DAPI). Finally, the sections were mounted with anti-fade mounting medium. The slides for BrdU staining were pretreated with solution containing 50% formamide, 280 mM NaCl, and 30 mM sodium citrate at 65°C for 2 h, incubated with 2 M HCl at 37°C for 30 min, and rinsed with 0.1 mol/L boric acid (pH 8.5) at room temperature for 10 min [2].

### Image Acquisition and Quantification

Fluorescent images were captured using a confocal laser scanning microscope (Olympus FV10i, Tokyo, Japan) with appropriate excitation wavelengths. The digital images were measured using Image-Pro Plus 6.0 software (Media Cybernetics, Rockville, MD, USA). In the statistical analysis, at least 6 representative fields from randomly-selected images were acquired from each sample by a blinded observer, and then the average cell count/integrated optical density values were calculated [23]. To measure the proportion of OPCs in different differentiation stages *in vitro*, each dish was scanned with a 20 × objective lens in 600 μm × 450 μm format in the *x–y* direction. The number of NG2/GFP, O4/GFP or CNPase/GFP double-positive cells among GFP-positive cells in each field was counted by a blinded observer [2].

### Primary Culture of Rat OPCs

The rat OPC proliferation culture was maintained as previously described [24, 25]. Isolated OPCs were plated at 10,000 cells/cm^2^ on poly-L-lysine-coated flasks and cultured in DMEM/F12 medium supplemented with 2% B27, 10 ng/mL platelet-derived growth factor AA (PDGF-AA, Gibco, Grand Island, NY, USA), and 10 g/mL basic fibroblast growth factor (bFGF, Gibco) for 3 days; then, the medium was replaced with DMEM/F12 medium without PDGF-AA and bFGF for 1 day to generate preOLs. For oligodendrocyte differentiation, 10% fetal bovine serum (FBS) was added to the preOL medium and cultured for 7 days. The medium was changed every 2 days.

### Quantitative Real-Time Polymerase Chain Reaction (qRT-PCR)

Genomic RNA was extracted with TRIzol reagent (Invitrogen, Carlsbad, CA, USA) according to the manufacturer’s instructions and as previously described [26]. The sense and antisense primers for GSK-3β were as follows: forward: 5′-TCCCTCAAATTAAGGCACATC-3′ and reverse: 5′-CACGGTCTCCAGTATTAGCATC-3′. The expression level of 18S ribosomal RNA (rRNA) served as an internal control for the samples, and was assessed using the following sense and antisense primers: forward: 5′-CCTGGATACCGCAGCTAGG A-3′ and reverse: 5′-GCGGCGCAATACGAATGCCCC-3′. The relative level of *Foxg1* mRNA expression was calculated according to the standard 2^−ΔΔCt^ method by normalization to the 18S rRNA mRNA level.

### Western Blot Analysis

Western blot analysis was performed as previously described [27, 28]. Total protein was extracted from cultured rat OPCs using cell lysis buffer supplemented with proteinase and phosphatase inhibitors. The nuclear proteins were extracted using a commercial kit (Thermo Fisher Scientific, Waltham, MA, USA). Protein samples were separated on 10% SDS-PAGE gels and transferred to nitrocellulose membranes. The primary antibodies were anti-Foxg1 (1:800, Sigma-Aldrich), anti-GSK-3β (1:600, Sigma-Aldrich), anti-β-actin (1:1000, Santa Cruz), anti-β-catenin (1:500, Abcam), and anti-histone H3 (1:1000, Sigma-Aldrich). The band intensity was quantified using ImageJ software (NIH, Bethesda, MD, USA). Values were normalized to the β-actin/histone H3 level.

### Lentiviral Vector Production

A lentivirus encoding small hairpin RNA (shRNA) for Foxg1 was designed and synthesized by GeneChem Co., Ltd (Shanghai, China). The *Foxg1* locus on chromosome 2 and its 200-bp flanking sequences were amplified by PCR from genomic rat DNA and inserted into the GV248 vector (the functional element is Ubi-EGFP-MCS-IRES-puromycin). The shRNA sequence for *Foxg1* was 5′-TCGGGCCAAGCTAGCCTTTAA-3′. To generate the construct, the scrambled sequence 5′-TTCTCCGAACGTGTCACGT-3′ was inserted. In the scrambled sequence, the nucleotides were randomly added with no target sequence tracks. Preparations of the recombinant lentivirus were made by transient co-transfection of HEK 293T cells accompanied by proper transfer vectors and lentiviral helper plasmids (20 μg of pGC-LV, 15 μg of pHelper 1.0, and 10 μg of pHelper 2.0) using Lipofectamine 2000 (Invitrogen, Waltham, MA, USA) according to the manufacturer’s instructions. OPCs were transfected with lentiviral *Foxg1* shRNA 72 h before experiments.

### EdU Labeling of OPCs

For EdU labeling, 3.5×10^4^ cells were seeded into each well of a 48-well plate in DMEM/F-12 medium (1:1) supplemented with 10% FBS. Twenty-four hours later, EdU (RiboBio, Guangzhou, China) was added to the medium at 10 μmol/L. Another 24 h later, the cells were fixed with 4% paraformaldehyde for 30 min. For EdU staining, the cells were then incubated with freshly-made 1× Apollo^®^567 reaction cocktail for 30 min at room temperature in the dark. Then, the cells were incubated in 0.5% Triton X-100 in PBS for 10 min at room temperature. After washing twice with PBS, the cells were fixed in methanol and then stained with Hoechst 33342. The percentage of EdU-positive cells was calculated as the number of Apollo^®^567-stained cells/the number of Hoechst 33342-stained cells.

### Statistical Analysis

Data were analyzed with SPSS software version 19.0 (IBM, Armonk, NY, USA) and are expressed as the mean ± SEM. *T*-tests or one-way analysis of variance (ANOVA) followed by the Newman-Keuls or Tukey’s HSD post tests were used for comparisons between two or multiple groups, respectively. Statistical significance was set at *P <* 0.05.

## Results

### Conditional Ablation of *Foxg1* Ameliorates Behavioral Deficits and Demyelination Induced by CPZ

**Fig. 1:**
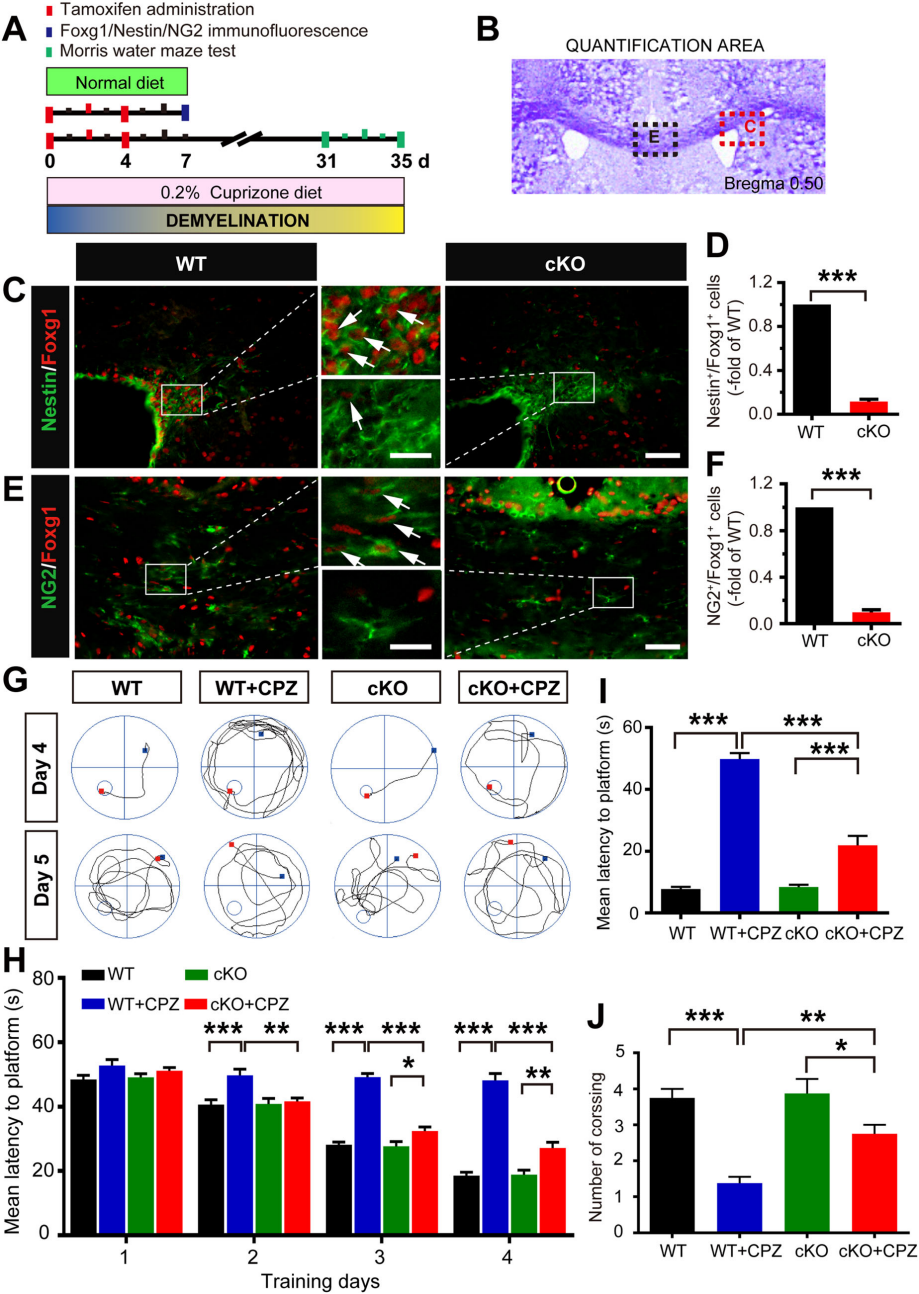
*Foxg1* cKO relieves CPZ-induced learning and memory impairments in mice. **A** Schematic of the cuprizone (CPZ) and tamoxifen (TM) administration time points during experiment. Adult 7-week-old mice were fed a diet containing 0.2% CPZ for 5 weeks (demyelination phase); TM was intraperitoneally injected (3 times, 24 h apart) from day 0 after feeding the CPZ diet; mice were sacrificed immediately after the Morris water maze (MWM) test. **B** Image showing the measured region in the coronal sections in **C**, **D** (red dashed box) and **E**, **F** (black dashed box). **C**, **D** Effect of *Foxg1* knockout on neural stem cells assessed by Nestin/Foxg1 immunofluorescence staining (**C**) and quantitative analysis (**D**). **E**, **F** Effect of *Foxg1* knockout on oligodendrocyte precursor cells assessed by NG2/Foxg1 immunofluorescence staining (**E**) and quantitative analysis (**F**). **G** Representative tracking from each group of mice in the MWM test on days 4 and 5 (small circles, location of the platform; blue and red points, start and end locations of the mouse, respectively). **H** Average latency to find a hidden platform during the first 4 days of training in the directional navigation experiment. **I** Latency to find the platform on day 5 when the platform was removed. **J** Number of crossings in the spatial exploration experiment on day 5 of the MWM (white arrows in **C** and **E**, representative double-labeled positive cells). *n* = 8 per group. Data are presented as the mean ± SEM. *P < 0.05, **P < 0.01, ***P < 0.001, Student’s *t*-test in **D**, **F**; one-way ANOVA with Tukey’s *post hoc* test in **H**–**J**. Scale bars, 25 μm in **C**, **E**; 10 μm in the enlarged images.

On the first day (day 0) of CPZ treatment, the mice were intraperitoneally injected with TM to induce *Foxg1* gene knockout (Fig. 1A). The results showed that, compared with control littermates, Foxg1 expression on neural stem cells in the subventricular zone (Fig. 1C, D) or OPCs in the corpus callosum region (Fig. 1E, F) was significantly reduced in *Foxg1*-cKO mice on day 7 after TM injection. Studies have shown that CPZ administration results in a decline in learning and memory in mice [29, 30]. The MWM test showed that *Foxg1* cKO significantly shortened the prolonged mean latency from day 2 to day 4 (Fig. 1G, H), and it significantly decreased the latency to the platform on day 5 (Fig. 1G, I). The crossing number of *Foxg1*-cKO mice was clearly increased compared with the WT+CPZ mice (Fig. 1J). These results suggested that *Foxg1* cKO significantly alleviates the learning and memory impairments induced by CPZ in mice.

**Fig. 2:**
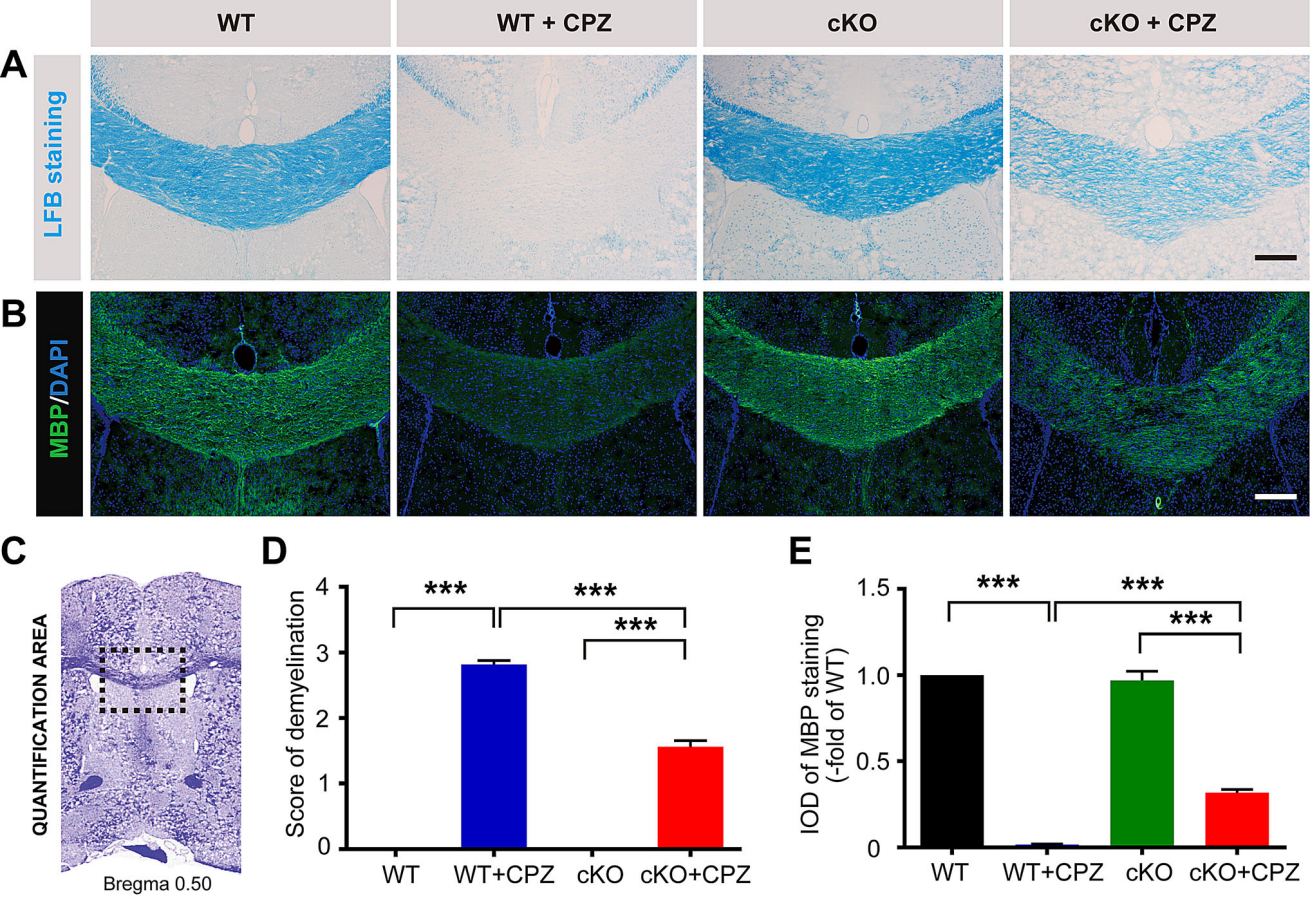
Conditional ablation of *Foxg1* alleviates demyelination induced by cuprizone (CPZ) in mice. **A** Representative images of LFB staining showing demyelination in the corpus callosum (CC) of each group. **B** Representative images of NG2 immunofluorescence staining in the CC of each group. **C** Image showing the measured region (black dashed box) in the coronal sections in **A**, **B**. and **D** The degree of demyelination was analyzed in sections as in **A**. **E** Quantification of the MBP fluorescence measured as integrated optical density (IOD) in each group from sections as in **B**. *n* = 8 per group; data are presented as the mean ± SEM; ***P < 0.001, one-way ANOVA with Tukey’s *post hoc* test; scale bars, 100 μm.

LFB staining showed that myelin was intact in the corpus callosum of WT mice, but it had almost entirely disappeared in the WT+CPZ mice (Fig. 2A). The demyelination score was significantly lower in the *Foxg1* cKO+CPZ group than in the WT+CPZ group (Fig. 2D). The statistical data from the myelin basic protein (MBP) immunofluorescence staining was consistent with the LFB staining results (Fig. 2B, E). These results indicated that demyelination induced by CPZ is significantly relieved by the absence of *Foxg1.*

### *Foxg1* cKO Decreases Proliferation but Promotes the Differentiation of OPCs in CPZ-Induced Demyelination

**Fig. 3:**
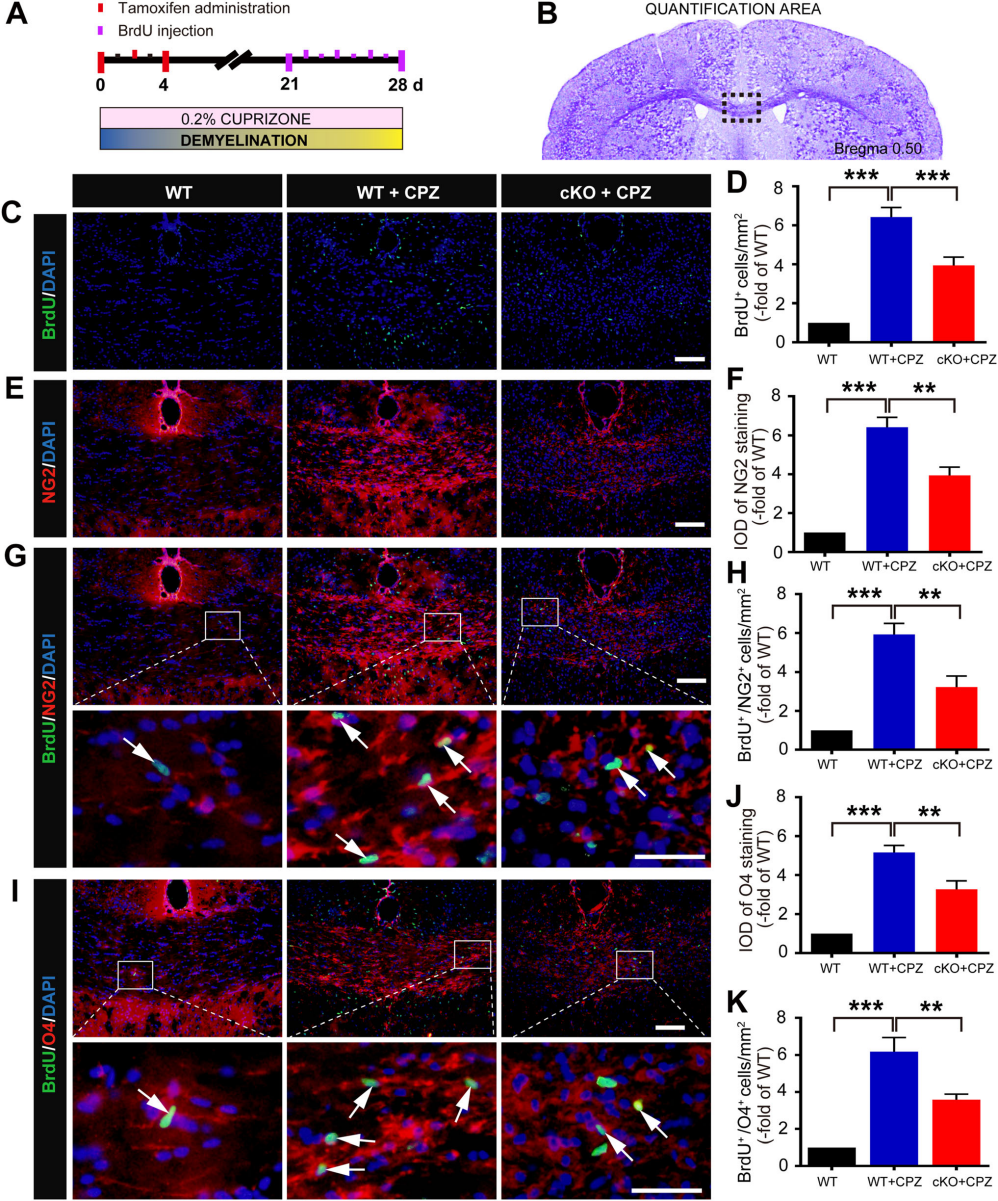
Conditional knockout of *Foxg1* decreases the proliferation of oligodendrocyte precursor cells in the corpus callosum (CC) of mice induced with cuprizone (CPZ). **A** Schematic of CPZ and BrdU administration time points during the experiment. Adult 7-week-old mice were fed a diet containing 0.2% CPZ for 4 weeks (demyelination phase) to assess the proliferation of intrinsic OPCs; Tamoxifen (TM) was intraperitoneally injected (3 times, 24 h apart) from day 0 after starting the CPZ diet; BrdU was intraperitoneally injected twice a day for 7 consecutive days, and then the mice were sacrificed. **B** Image showing the measured region (black dashed box) in coronal sections as in **C**–**K**. **C** Immunofluorescence staining of BrdU in the CC of each group. **D** Quantitative analysis of BrdU^+^ cells as in **C**. **E** Immunofluorescence staining of NG2 in the CC of each group. **F** Quantitative analysis of NG2^+^ cells as in **E**. **G**, **H** Immunofluorescence staining of BrdU/NG2 (**G**) and quantitative analysis (**H**) in the CC of each group. **I** Immunofluorescence staining of BrdU/O4 in the CC of each group. **J**, **K** Quantitative analysis of O4^+^ cells (**J**) and BrdU^+^/O4^+^ cells as in **I**. White arrows, representative double-labeled positive cells; *n* = 8 per group; data are presented as the mean ± SEM; **P < 0.01, ***P < 0.001, one-way ANOVA with Tukey’s *post hoc* test; scale bars, 50 μm in **C**, **E** and the upper panels in **G**, **I**; 20 μm in the lower panels of **G**, **I**.

**Fig. 4:**
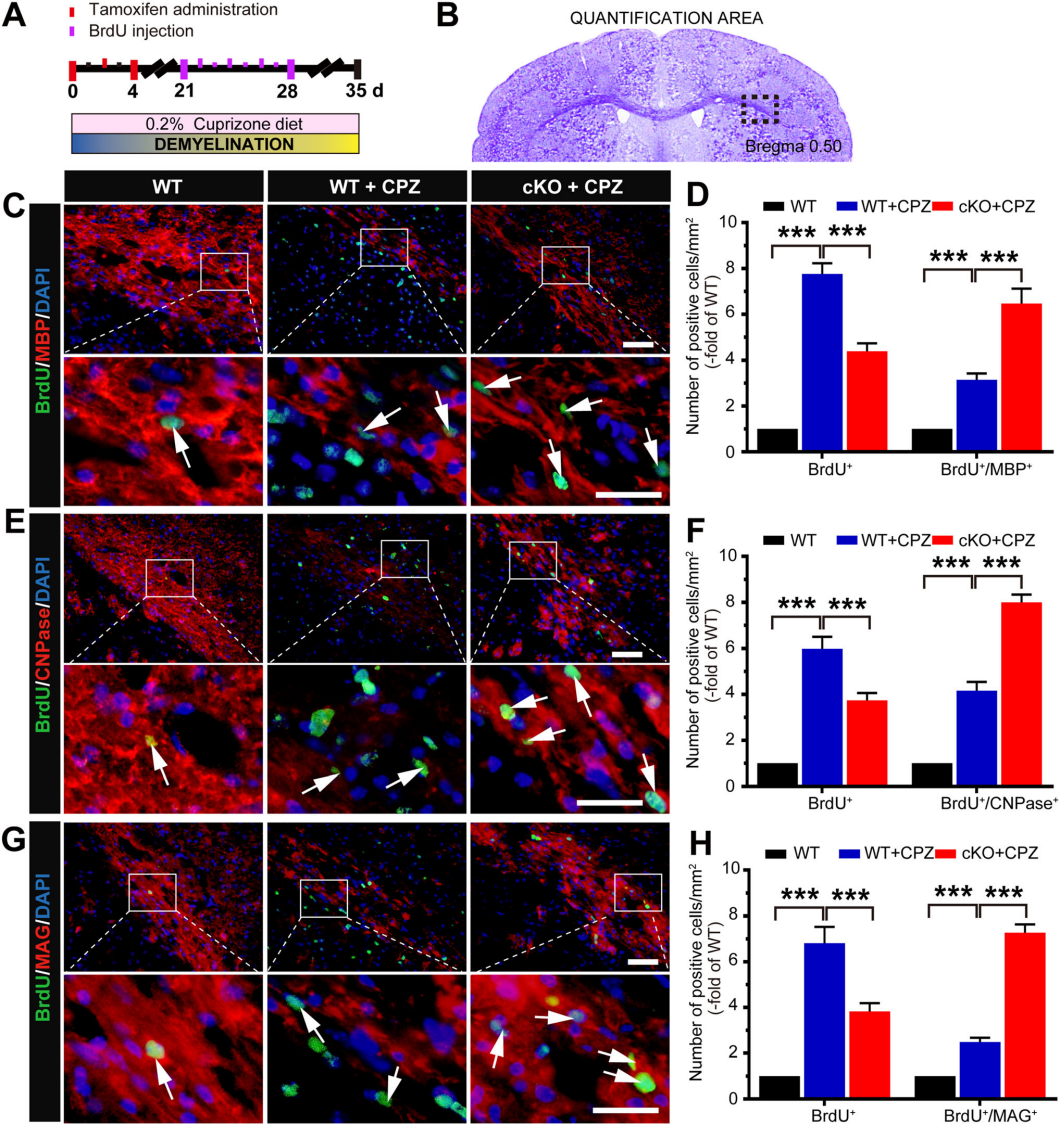
Conditional knockout of *Foxg1* promotes the differentiation of oligodendrocyte precursor cells in the corpus callosum (CC) of mice induced by CPZ. **A** Schematic of cuprizone (CPZ) and BrdU administration time points during the experiment. Adult 7-week-old mice were fed a diet containing 0.2% CPZ for 5 weeks (demyelination phase); Tamoxifen (TM) was intraperitoneally injected (3 times, 24 h apart) from day 0 after feeding on a CPZ diet; BrdU was intraperitoneally injected twice a day for 7 consecutive days, and the mice were sacrificed on day 35. **B** Image showing the measured region (black dashed box) in coronal sections as in **C**–**H**. **C**–**H** Immunofluorescence double-labeled staining and quantitative analysis of BrdU/MBP (**C**, **D**), BrdU/CNPase (**E**, **F**), and BrdU/MAG (**G**, **H**) in the CC of *Foxg1-*cKO mice. White arrows indicate representative double-labeled positive cells; *n* = 8 per group; data are presented as the mean ± SEM; **P < 0.01, ***P < 0.001, one-way ANOVA with Tukey’s *post hoc* test; scale bars, 20 μm in the upper panel; 10 μm in the lower panel.

BrdU labeling was used to assess cell proliferation (Fig. 3A) and differentiation (Fig. 4A) in the corpus callosum, and the results showed that the number of BrdU-positive cells was significantly lower in the *Foxg1* cKO+CPZ group than the WT+CPZ group (Fig. 3C, D). Furthermore, the increase in percentage of NG2^+^ cells (Fig. 3E, F) and O4^+^ cells (Fig. 3J) cells in the WT+CPZ group was prevented by *Foxg1*-cKO treatment. The number of proliferating OPCs, indicated by NG2^+^/BrdU^+^ (Fig. 3G, H) or BrdU^+^/O4^+^ (Fig. 3I, K), was also decreased; however, the number of new mature oligodendrocytes labeled with BrdU^+^/MBP^+^ (Fig. 4C, D), BrdU^+^/CNPase^+^ (Fig. 4E, F), and BrdU^+^/MAG^+^ (Fig. 4G, H) differentiated from the OPCs was significantly higher in the *Foxg1* cKO+CPZ group than in the WT+CPZ group. These data suggested that *Foxg1* cKO decreases the production or proliferation of OPCs but promotes their differentiation.

### *Foxg1* cKO Facilitates Myelination and the Recovery of Cognitive Function During Remyelination

**Fig. 5:**
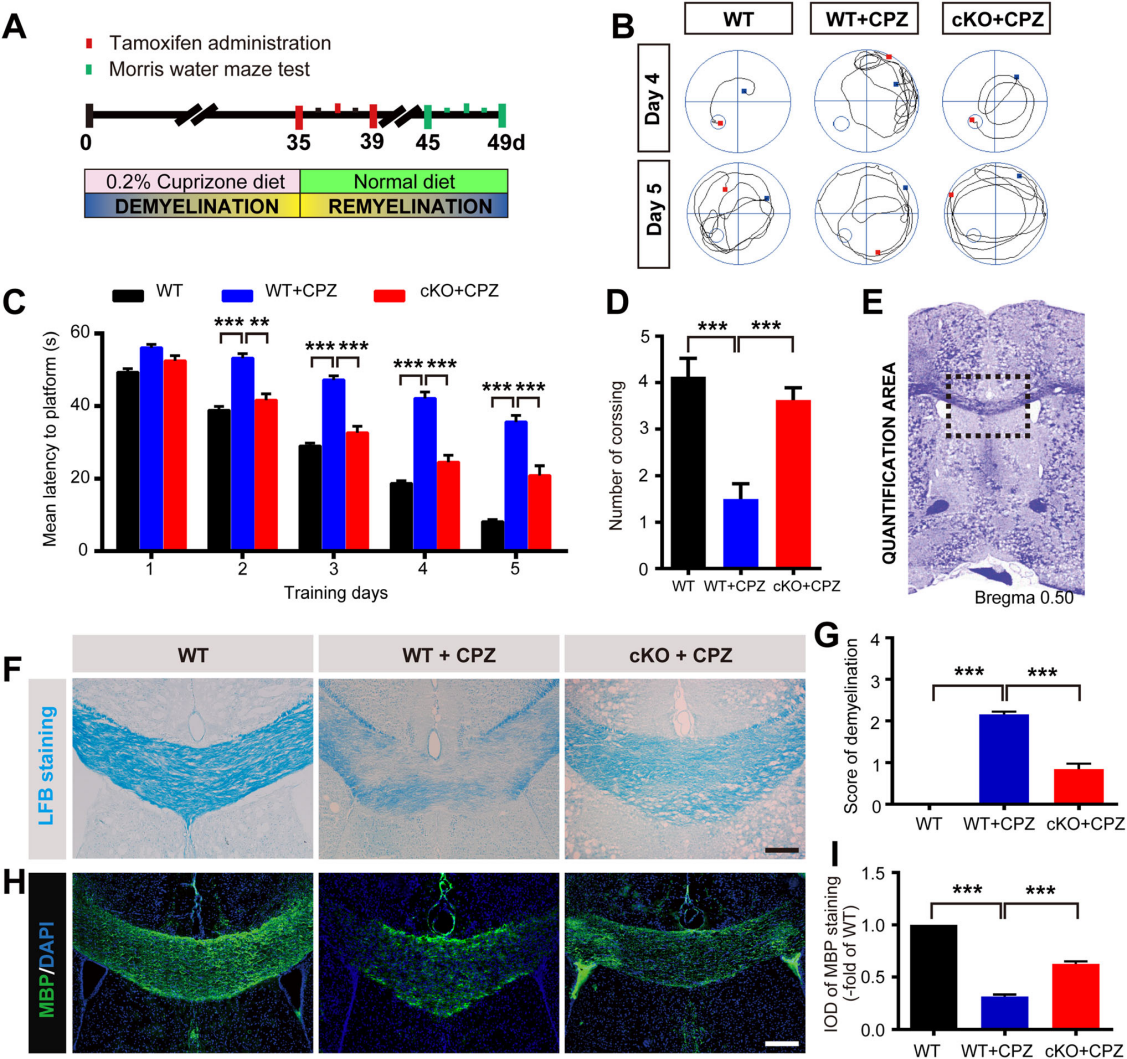
*Foxg1* cKO promotes the recovery of cognitive function and remyelination after CPZ withdrawal in mice. **A** Timeline of the experimental design. Adult 7-week-old mice were fed a diet containing 0.2% cuprizone (CPZ) for 5 weeks (demyelination phase), followed by a normal diet for 2 weeks (remyelination phase); Tamoxifen (TM) was intraperitoneally injected (3 times, 24 h apart) after CPZ withdrawal; mice were sacrificed immediately after the Morris water maze (MWM) test. **B** Representative tracking in each group in the MWM test on days 4 and 5 (small circles, location of the platform; blue and red points, start and end locations, respectively). **C** Average latency for each group to find a hidden platform over the first 4 days of training in the directional navigation experiment and on day 5 when the platform was removed. **D** Number of crossings in each group in the spatial exploration experiment on day 5 of the MWM. **E** Image showing the measured region (black dashed box) in coronal sections as in **F**–**I**. **F** Representative images of LFB staining for demyelination in the corpus callosum in each group. **G** Analysis of the degree of demyelination as in **F**. **H**, **I** MBP immunofluorescence (**H**) and analysis of integrated optical density (IOD) (**I**) in each group. *n* = 8 per group; data are presented as the mean ± SEM; **P < 0.01, ***P < 0.001, one-way ANOVA with Tukey’s *post hoc* test; scale bars, 50 μm.

To study the role of *Foxg1* in the regeneration of OLs after CPZ-induced myelin sheath injury, TM was administered on day 1 of CPZ withdrawal (Fig. 5A). Behavioral results showed that the average latency was significantly longer in the WT+CPZ than the WT group; however, *Foxg1* deletion in CPZ-treated mice clearly decreased the latency (Fig. 5B, C). In the spatial exploration experiment on day 5, the number of platform crossings was significantly higher in the *Foxg1* cKO+CPZ than the WT+CPZ group (Fig. 5D). These results suggested that *Foxg1* cKO significantly promotes the recovery of learning and memory after CPZ withdrawal in mice.

After 2 weeks of feeding without CPZ, the demyelination score was decreased (Fig. 5F, G) and the fluorescence intensity of MBP significantly enhanced (Fig. 5H, I) in the *Foxg1*-cKO mice. These results indicated that *Foxg1* deficiency favors myelin repair after CPZ-induced demyelinating injury.

### *Foxg1* cKO Inhibits Proliferation but Promotes the Differentiation of OPCs *In Vitro*

**Fig. 6:**
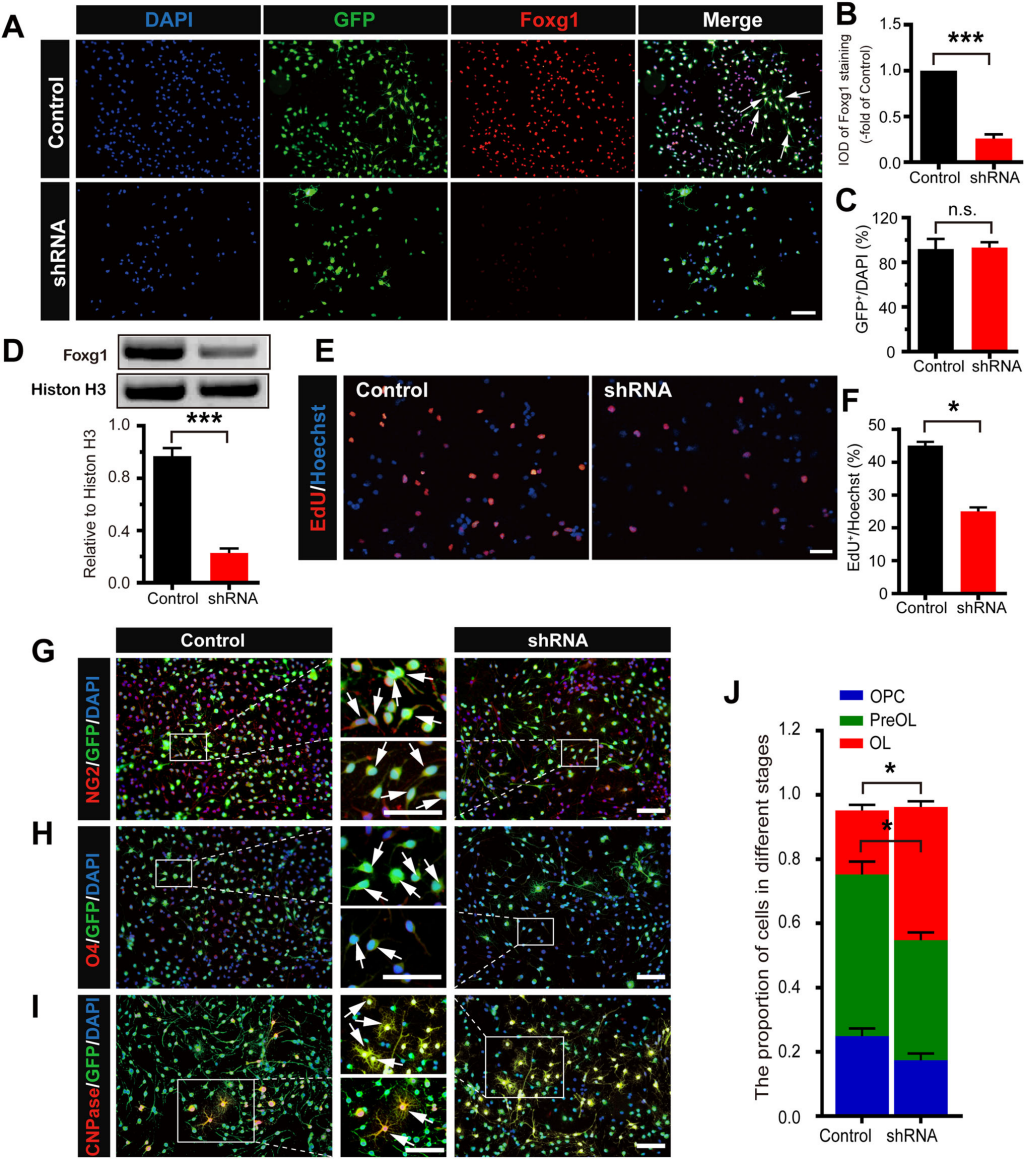
Knockdown of *Foxg1* decreases proliferation while promoting the differentiation of OPCs *in vitro*. **A** *Foxg1* knockdown decreases the expression of Foxg1 in primary cultured rat OPCs. Cells transfected with a lentiviral GFP-tagged vector inserted into a scrambled sequence (Control) or lentiviral GFP-tagged *Foxg1* shRNA (shRNA) for 3 days and immunostained with anti-Foxg1 antibody. **B** Quantification of Foxg1 fluorescence integrated optical density (IOD) as in **A**. **C** The transfection efficiency as defined by GFP fluorescence. **D** Western blots and quantitative analyses of Foxg1 protein expression in primary cultured rat OPCs. **E**, **F** Representative images of EdU-positive proliferating cells (**E**) and quantitative analysis (**F**) of Control and *Foxg1* shRNA-treated OPCs. **G**–**I** Representative images of NG2 (**G**), O4 (**H**), and CNPase (**I**) staining in each group of cells. **J** Cell proportions at different stages during the differentiation of OPCs. White arrows, representative double-labeled positive cells; *n* = 6 per group; data are presented as the mean ± SEM; *P < 0.05, ***P < 0.001, n.s. not significant, Student’s *t*-test; scale bars, 40 μm.

*In vitro*, GFP-tagged shRNA knockdown of *Foxg1* using lentiviral transfection was required to confirm its function in the proliferation and differentiation of cultured OPCs. The results of immunofluorescence staining (Fig. 6A, B) and Western blotting (Fig. 6D) demonstrated that *Foxg1* knockdown strongly decreased the expression of Foxg1 in primary cultured rat OPCs. The lentiviral transfection efficiency was defined by GFP fluorescence (Fig. 6C). The results of EdU staining showed that the number of EdU^+^ cells was significantly smaller in the *Foxg1* shRNA than the control group (Fig. 6E, F). These results indicated that, to a certain extent, knockdown of *Foxg1* decreases the proliferation of OPCs *in vitro*, suggesting that *Foxg1* participates in the regulation of OPC proliferation.

To confirm whether *Foxg1* regulates OPC differentiation, we downregulated the level of *Foxg1* in OPCs and analyzed the number of immature and mature OLs. In contrast to the control group, most of the cells in the shRNA group were labeled with CNPase (Fig. 6I), and only a few were labeled with NG2 (Fig. 6G) or O4 (Fig. 6H). The number of O4-positive cells was significantly lower and CNPase-positive cells significantly higher in the *Foxg1* shRNA than in the Ctrl group (Fig. 6J), suggesting that *Foxg1* plays a negative regulatory role during OPC differentiation into OLs.

### *Foxg1* May Participate in the Proliferation and Differentiation of OPCs Through the Wnt Signaling Pathway

**Fig. 7:**
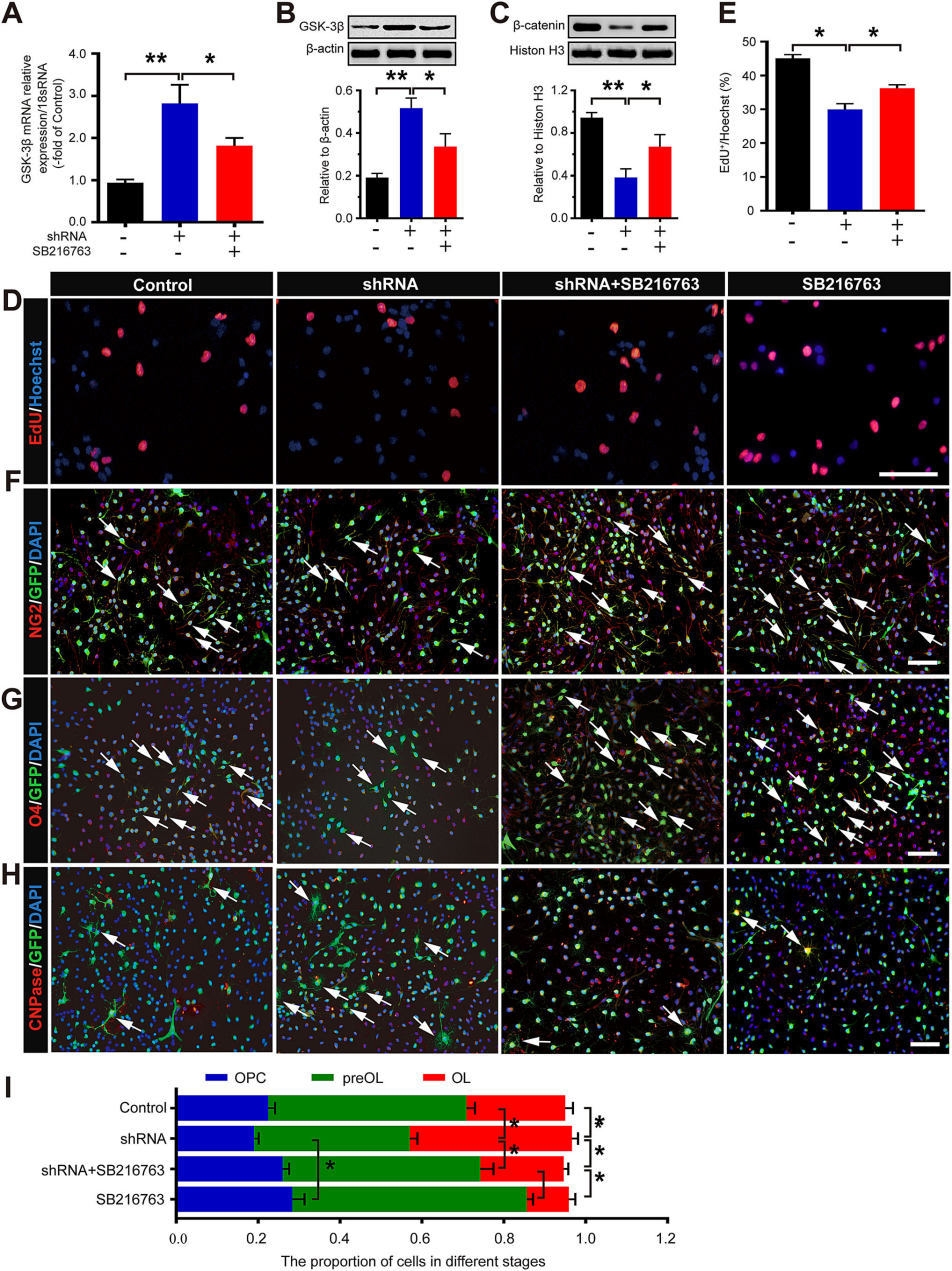
*Foxg1* regulates the proliferation and differentiation of OPCs by the Wnt signaling pathway. **A** GSK-3β mRNA expression in each group of cells assessed by qRT-PCR. **B**, **C** Western blots and quantitative analyses of the protein expression of GSK-3β (**B**) and nuclear β-catenin (**C**) in each group of cells. **D**, **E** EdU staining (**D**) and quantitative analysis (**E**) in each group of cells. **F**, **G** Representative images of NG2 (**F**) and O4 (**G**) immunofluorescence staining in each group of cells. **H** Representative images of CNPase immunofluorescence staining in each group of cells cultured in DMEM/F12 medium containing 10% FBS without PDGF-AA and bFGF for 7 days. **I** Cell proportions at different stages during the differentiation of OPCs. White arrows, representative double-labeled positive cells; *n* = 6 for each group; data are presented as the mean ± SEM; *P < 0.05, **P < 0.01, one-way ANOVA with Tukey’s *post hoc* test; scale bars, 40 µm.

To explore whether *Foxg1* influences Wnt signal activation, we assessed the expression of GSK-3β and β-catenin. The results showed that the level of GSK-3β mRNA (Fig. 7A) and protein (Fig. 7B) was significantly higher in the *Foxg1* shRNA than in the control group, while the expression of β-catenin was significantly decreased in *Foxg1-*knockdown OPCs (Fig. 7C). The GSK-3β inhibitor SB216763 reversed the decrease in β-catenin induced by *Foxg1* knockdown (Fig. 7C). Moreover, SB216763 treatment also clearly increased the number of EdU^+^ cells (Fig. 7D, E) and the proportions of NG2^+^ (Fig. 7F, I) and O4^+^ cells (Fig. 7G, I) and decreased the proportion of CNPase (Fig. 7H, I) when compared with the *Foxg1* shRNA group. These results indicated that *Foxg1* might be involved in the proliferation and differentiation of OPCs through the Wnt signaling pathway.

## Discussion

As described in previous reports, *Foxg1* is abundantly expressed in the CNS [31] and plays pivotal roles in organogenesis through the regulation of proliferation and the specification of cell fate [32]. However, the role of *Foxg1* in the oligodendroglial lineage and the underlying mechanisms remain unclear. Therefore, the present study was designed to determine the effect of *Foxg1* and the mechanisms underlying the regulation of the Wnt signaling pathway following CPZ-induced demyelination.

CPZ is a copper chelator that impacts cell metabolism and leads to oligodendrocyte death and demyelination accompanied by weight loss and behavioral disorders [33]. The CPZ-fed C57BL/6 mouse model has been increasingly used to study demyelination and remyelination in the CNS [33, 34]. The present results are consistent with previous reports [35, 36], demonstrating that we successfully replicated the CPZ-induced demyelination model in the ICR strain of mice [37]. However, for the first time, we found that conditional knockout of *Foxg1* in NSPCs reversed the demyelination and spatial learning and memory impairments; moreover, a more evident remyelination was found after CPZ withdrawal. It has been reported that different subcellular localizations of *Foxg1* control the machinery that causes cell differentiation, replication, and bioenergetics. A fraction of *Foxg1* is recruited to mitochondria and plays a major role in neuronal survival, differentiation, and plasticity [38]. In addition, dietary triheptanoin rescues oligodendrocyte loss in a mouse model of Canavan disease [39], as well as the functional and molecular abnormalities in *Foxg1*^+/−^ mice [40]. Thus, it should be noted that the food used in our study was a normal diet. To induce demyelination, 0.2% (w/w) CPZ was mixed into ground standard rodent chow. In addition, each group of mice received the same amount of chow. Thus, we confirmed that *Foxg1* might participate in the repair of myelin sheath damage in mice with CPZ-induced demyelination.

Oligodendrocytes are derived from OPCs [41], which populate the CNS starting from embryonic development [42, 43]. Previous studies [44, 45] have demonstrated that demyelinating injury induces OPC proliferation; however, studies on the differentiation of OPCs into mature oligodendrocytes and remyelination are limited. The mechanisms underlying the effect of *Foxg1* on the regulation of demyelination and remyelination remain to be clarified. Here, we found that CPZ administration increased the number of OPCs, which indicated that CPZ injury initiates the endogenous repair process and promotes the production of OPCs from neural stem cells of the subventricular zone region and migration to the injured area [46]. In the CPZ-induced demyelinated mouse corpus callosum, OPC proliferation was decreased by *Foxg1* cKO. Moreover, double labeling using BrdU and cell-specific markers showed that *Foxg1* cKO promoted OPC differentiation into mature OLs. Previous research has demonstrated that *Foxg1* may be necessary for maintenance of the NSC pool in the CNS and that genetic inactivity of *Foxg1* promotes both gliogenesis and neurogenesis [11]. NSCs are maintained in a quiescent state in the adult CNS [47]. Although it has been reported that *Foxg1* cKO in nestin-positive NSCs promotes neurogenesis and gliogenesis [11], it did not markedly change the myelin sheath and behavior in mice fed a normal diet in our study. We conclude that the change was so mild that there was no marked qualitative difference between the groups. However, the CPZ diet induced very serious demyelination accompanied by a change in the stem cell niche and OPC proliferation, and under this pathological situation, *Foxg1* cKO clearly promoted OPC differentiation. *In vitro*, purified primary OPCs were used to assess the effects of *Foxg1* inhibition on OPC proliferation and differentiation. It has been demonstrated that the loss of *Foxg1* leads to cycle exit and promotes the differentiation of premature cells [48, 49]. Consistent with previous reports [9–11], *Foxg1* knockdown inhibited the proliferation and promoted the differentiation of OPCs under normal culture conditions. As a result, the number of OPCs in the CPZ-treated corpus callosum area was reduced due to accelerated differentiation. The differentiated mature OLs encapsulated the axons of the neurons to form new myelin sheaths, thereby alleviating the myelin damage caused by CPZ or accelerating remyelination.

The Wnt/β-catenin signaling pathway plays an important role in regulating cell proliferation and differentiation [50–52]. Studies have shown that inhibition of GSK-3β expression in the Wnt signaling pathway inhibits the differentiation of OPCs while promoting their proliferation [53, 54]. Here, we found a significant increase in GSK-3β at the mRNA and protein levels in OPCs after *Foxg1* knockdown. We used 10 μmol/L SB216763 to inhibit the function of GSK-3β [55, 56], and found that this treatment reversed the effect of *Foxg1* on OPC proliferation and differentiation and the nuclear level of β-catenin. These results are in agreement with reports in the literature [15]. Together with the results of the *in vivo* experiments performed here, it can be concluded that conditional knockout or knockdown of *Foxg1* effectively facilitates the differentiation of OPCs into OLs and promote the recovery of damaged myelin, and the Wnt/β-catenin signaling pathway is involved in this process. Given the increasing importance of glia-glia crosstalk and the inflammatory microenvironment in demyelination or remyelination [57–59], future studies are needed to focus on the microglia, astrocytes, and even the interaction between microglia, astrocytes, and oligodendrocytes, to further clarify the role of the Wnt/β-catenin signaling pathway in CPZ-induced demyelination or remyelination.

In summary, the present study provides evidence that, in CPZ-induced demyelinating mice, *Foxg1* knockout inhibits the proliferation but promotes the differentiation of OPCs into mature OLs by regulating the activity of the Wnt/β-catenin signaling pathway, which ultimately reduces the severity of demyelination and promotes remyelination. Therefore, *Foxg1* might be a promising novel target to enhance endogenous remyelination in patients with demyelinating diseases such as MS. Further experiments are required to investigate the precise targets of *Foxg1* in the Wnt/β-catenin signaling pathway and its interactions with other types of glial cells involved in demyelinating diseases in the CNS.
